# [μ-1,1′-(Butane-1,4-di­yl)di-1*H*-benz­imidazole-κ^2^ *N* ^3^:*N* ^3′^]bis­{[*N*,*N*′-bis(car­boxy­meth­yl)ethyl­enediamine-*N*,*N*′-di­acetato-κ^5^ *O*,*O*′,*O*′′,*N*,*N*′]mercury(II)} methanol disolvate

**DOI:** 10.1107/S1600536809026221

**Published:** 2009-07-11

**Authors:** Xue-Wen Zhu, Bo Xiao, Zhi-Gang Yin, Heng-Yu Qian, Gang-Sen Li

**Affiliations:** a Key Laboratory of Surface and Interface Science of Henan, School of Materials & Chemical Engineering, Zhengzhou University of Light Industry, Zhengzhou 450002, People’s Republic of China

## Abstract

The binuclear title complex, [Hg_2_(C_10_H_14_N_2_O_8_)_2_(C_18_H_18_N_4_)]·2CH_3_OH, lies on an inversion center with the unique Hg^II^ ion coordinated in a disorted octa­hedral environment with one Hg—N bond significantly shorter than the other two. In the crystal structure, inter­molecular O—H⋯O hydrogen bonds link complex and solvent mol­ecules into a three-dimensional network.

## Related literature

For the synthesis, see: Xiao *et al.* (2004 ▶); Xie *et al.* (2002 ▶). For bond lengths related mercury compounds, see: Guo & Dong (2009 ▶); Aghabozorg, *et al.* (2008 ▶).
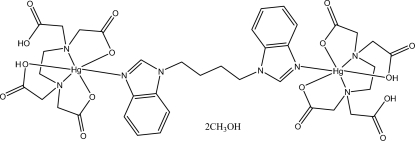


## Experimental

### 

#### Crystal data

[Hg_2_(C_10_H_14_N_2_O_8_)_2_(C_18_H_18_N_4_)]·2CH_4_O
*M* *_r_* = 1336.09
Monoclinic, 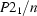

*a* = 10.274 (2) Å
*b* = 19.990 (3) Å
*c* = 11.4717 (17) Å
β = 104.035 (13)°
*V* = 2285.7 (7) Å^3^
*Z* = 2
Mo *K*α radiation
μ = 6.79 mm^−1^
*T* = 291 K
0.24 × 0.20 × 0.18 mm

#### Data collection

Bruker SMART APEX diffractometer
Absorption correction: multi-scan (*SADABS*; Sheldrick, 1996 ▶) *T*_min_ = 0.21, *T*_max_ = 0.29
14826 measured reflections
4397 independent reflections
3744 reflections with *I* > 2σ(*I*)
*R*_int_ = 0.062

#### Refinement

*R*[*F* ^2^ > 2σ(*F* ^2^)] = 0.045
*wR*(*F* ^2^) = 0.121
*S* = 1.04
4397 reflections
310 parameters
H-atom parameters constrained
Δρ_max_ = 1.08 e Å^−3^
Δρ_min_ = −1.60 e Å^−3^

### 

Data collection: *SMART* (Bruker, 1998 ▶); cell refinement: *SAINT* (Bruker, 1999 ▶); data reduction: *SAINT*; program(s) used to solve structure: *SHELXTL* (Sheldrick, 2008 ▶); program(s) used to refine structure: *SHELXTL*; molecular graphics: *SHELXTL* and *PLATON* (Spek, 2009 ▶); software used to prepare material for publication: *SHELXTL*.

## Supplementary Material

Crystal structure: contains datablocks I, global. DOI: 10.1107/S1600536809026221/lh2830sup1.cif

Structure factors: contains datablocks I. DOI: 10.1107/S1600536809026221/lh2830Isup2.hkl

Additional supplementary materials:  crystallographic information; 3D view; checkCIF report

## Figures and Tables

**Table 1:** Selected bond lengths (Å)

Hg1—N1	2.138 (6)
Hg1—N4	2.364 (6)
Hg1—N3	2.390 (6)
Hg1—O3	2.473 (5)
Hg1—O5	2.547 (5)
Hg1—O1	2.604 (6)

**Table 2:** Hydrogen-bond geometry (Å, °)

*D*—H⋯*A*	*D*—H	H⋯*A*	*D*⋯*A*	*D*—H⋯*A*
O2—H2⋯O5^i^	0.82	1.74	2.534 (7)	164
O8—H8⋯O4^ii^	0.82	1.84	2.462 (7)	131
O9—H9⋯O6^iii^	0.82	2.00	2.744 (7)	150

Symmetry codes: (i) 
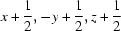
; (ii) 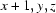; (iii) 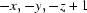.

**Fig. 1.**
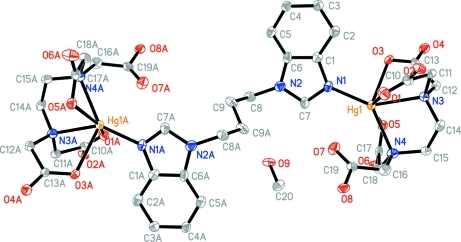
The molecular structure with ellipsoids shown at the 30% level. Symmetry code: (A) 1-x, 2-y, -z. The symmetry related solvent methanol molecule is not shown.

**Fig. 2.**
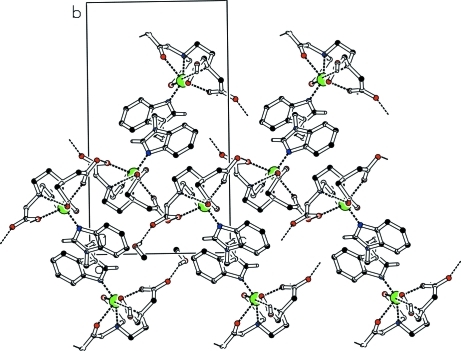
Part of the crystal structure with hydrogen bonds shown as dashed lines. Only H atoms included in the hydrogen bonds have been included.

## supplementary crystallographic information

### Comment

Research of transition metal-organic polymers has been rapidly expanding because of their intriguing topologies and potential applications in catalysis, fluorescence, electrical conductivity and magnetism. As a result, the preparation of coordination polymers with fascinating frameworks has attracted considerable attention in recent years. For our group, metal-organic nonlinear optical (NLO) materials are of interest for various applications such as optical data processing and biological imaging (Xiao *et al.*,  2004). Some mercury complexes have already been synthesised (Guo, *et al.*, 2009; Aghabozorg *et al.*, 2008), and herein we present the synthesis and crystal structure of the title complex (I) using EDTA and bbbm (Xie, *et al.*, 2002) as ligands. The molecular structure of the title complex is shown in Fig. 1. The dinuclear complex lies on an inversion center with the unique Hg^II^ ion coordinated in a disorted octahedral coordination environment with one Hg—N bond significantly shorter than the other two, most likely, in part, as a results of steric effects from the bulky ligands. The Hg-O bond lengths are in agreement with those found in related Hg(II) complexes (Guo & Dong, 2009; Aghabozorg, *et al.,* 2008). The intramolecular Hg···Hg distance is ca. 12.21Å. In the crystal structure, intermolecular O-H···O hydrogen bonds link complex and solvent molecules into a three-dimensional network (Fig. 2). In addition there are weak π···π stacking interactions between benzimidazole rings related by inversion symmetry with a centroid to centroid distance of 3.556 (4)Å.

### Experimental

Methanol solutions of HgCl_2_.2H_2_O (76 mg, 0.2 mmol), Na_2_edta (67 mg, 0.2 mmol) and 1, 1'-(1,4-butylidene)bis-1*H*-benzimidazole (15 mg, 0.1 mmol) were mixed in a 2:2:1 molar ratio, and the reaction mixture was stirred at about 300 K for 2 h. Colourless crystals ofthe title compound were obtained from the solution after three weeks at room temperature.

### Refinement

H atoms were placed in calculated positions with O-H = 0.82Å and C-H = 0.93-0.98Å and included in calculated postions with U_iso_(H) = 1.2U_eq_(C) or 1.5U_eq_(C) for methyl H atoms.

### Crystal data

**Table:**

[Hg_2_(C_10_H_14_N_2_O_8_)_2_(C_18_H_18_N_4_)]·2CH_4_O	*F*(000) = 1308
*M**_r_* = 1336.09	*D*_x_ = 1.941 Mg m^−^^3^
Monoclinic, *P*2_1_/*n*	Mo *K*α radiation, λ = 0.71073 Å
Hall symbol: -P 2yn	Cell parameters from 2608 reflections
*a* = 10.274 (2) Å	θ = 2.4–25.3°
*b* = 19.990 (3) Å	µ = 6.79 mm^−^^1^
*c* = 11.4717 (17) Å	*T* = 291 K
β = 104.035 (13)°	Prism, colorless
*V* = 2285.7 (7) Å^3^	0.24 × 0.20 × 0.18 mm
*Z* = 2	

### Data collection

**Table:**

Bruker SMART APEX diffractometer	4397 independent reflections
Radiation source: sealed tube	3744 reflections with *I* > 2σ(*I*)
graphite	*R*_int_ = 0.062
φ and ω scans	θ_max_ = 26.0°, θ_min_ = 2.9°
Absorption correction: multi-scan (*SADABS*; Sheldrick, 1996)	*h* = −12→12
*T*_min_ = 0.21, *T*_max_ = 0.29	*k* = −23→24
14826 measured reflections	*l* = −14→13

### Refinement

**Table:**

Refinement on *F*^2^	Secondary atom site location: difference Fourier map
Least-squares matrix: full	Hydrogen site location: inferred from neighbouring sites
*R*[*F*^2^ > 2σ(*F*^2^)] = 0.045	H-atom parameters constrained
*wR*(*F*^2^) = 0.121	*w* = 1/[σ^2^(*F*_o_^2^) + (0.08*P*)^2^ + 1.99*P*] where *P* = (*F*_o_^2^ + 2*F*_c_^2^)/3
*S* = 1.04	(Δ/σ)_max_ < 0.001
4397 reflections	Δρ_max_ = 1.08 e Å^−^^3^
310 parameters	Δρ_min_ = −1.60 e Å^−^^3^
0 restraints	Absolute structure: Refinement
Primary atom site location: structure-invariant direct methods	

### Special details

**Table:**

Geometry. All e.s.d.'s (except the e.s.d. in the dihedral angle between two l.s. planes) are estimated using the full covariance matrix. The cell e.s.d.'s are taken into account individually in the estimation of e.s.d.'s in distances, angles and torsion angles; correlations between e.s.d.'s in cell parameters are only used when they are defined by crystal symmetry. An approximate (isotropic) treatment of cell e.s.d.'s is used for estimating e.s.d.'s involving l.s. planes.
Refinement. Refinement of *F*^2^ against ALL reflections. The weighted *R*-factor *wR* and goodness of fit *S* are based on *F*^2^, conventional *R*-factors *R* are based on *F*, with *F* set to zero for negative *F*^2^. The threshold expression of *F*^2^ > σ(*F*^2^) is used only for calculating *R*-factors(gt) *etc*. and is not relevant to the choice of reflections for refinement. *R*-factors based on *F*^2^ are statistically about twice as large as those based on *F*, and *R*- factors based on ALL data will be even larger.

### Fractional atomic coordinates and isotropic or equivalent isotropic displacement parameters (Å^2^)

**Table:**

	*x*	*y*	*z*	*U*_iso_*/*U*_eq_	
C1	0.1031 (7)	0.0816 (3)	1.0464 (6)	0.0335 (15)	
C2	0.0283 (8)	0.1117 (4)	1.1206 (6)	0.0395 (17)	
H2A	−0.0216	0.1506	1.0997	0.047*	
C3	0.0363 (8)	0.0778 (4)	1.2276 (7)	0.0421 (17)	
H3	−0.0152	0.0924	1.2791	0.051*	
C4	0.1222 (7)	0.0205 (4)	1.2612 (6)	0.0357 (15)	
H4	0.1289	0.0008	1.3359	0.043*	
C5	0.1937 (7)	−0.0058 (4)	1.1866 (6)	0.0370 (16)	
H5	0.2490	−0.0428	1.2085	0.044*	
C6	0.1803 (7)	0.0253 (4)	1.0767 (7)	0.0379 (16)	
C7	0.1798 (7)	0.0552 (4)	0.8889 (7)	0.0419 (17)	
H7	0.1937	0.0537	0.8118	0.050*	
C8	0.3271 (7)	−0.0452 (4)	0.9728 (7)	0.0393 (16)	
H8A	0.3203	−0.0567	0.8894	0.047*	
H8B	0.3010	−0.0842	1.0121	0.047*	
C9	0.4721 (7)	−0.0270 (4)	1.0330 (7)	0.0414 (18)	
H9A	0.4770	−0.0121	1.1144	0.050*	
H9B	0.5275	−0.0667	1.0376	0.050*	
C10	0.0718 (8)	0.3206 (4)	0.9820 (7)	0.0406 (17)	
C11	−0.0801 (8)	0.3230 (3)	0.9266 (7)	0.0349 (16)	
H11A	−0.1279	0.3025	0.9806	0.042*	
H11B	−0.1098	0.3690	0.9130	0.042*	
C12	−0.2518 (7)	0.2659 (4)	0.7742 (7)	0.0370 (15)	
H12A	−0.2759	0.2549	0.6893	0.044*	
H12B	−0.3076	0.3029	0.7873	0.044*	
C13	−0.2778 (7)	0.2037 (4)	0.8490 (6)	0.0321 (15)	
C14	−0.0796 (8)	0.3303 (3)	0.7160 (6)	0.0348 (15)	
H14A	0.0002	0.3564	0.7497	0.042*	
H14B	−0.1539	0.3611	0.6894	0.042*	
C15	−0.0589 (8)	0.2912 (4)	0.6100 (7)	0.0385 (16)	
H15A	−0.1428	0.2700	0.5699	0.046*	
H15B	−0.0341	0.3218	0.5534	0.046*	
C16	0.0334 (8)	0.1861 (3)	0.5512 (7)	0.0356 (15)	
H16A	0.0232	0.2068	0.4731	0.043*	
H16B	0.1149	0.1595	0.5675	0.043*	
C17	−0.0798 (7)	0.1425 (3)	0.5481 (6)	0.0313 (14)	
C18	0.1874 (7)	0.2721 (4)	0.6685 (7)	0.0415 (17)	
H18A	0.2052	0.2850	0.5923	0.050*	
H18B	0.1872	0.3124	0.7154	0.050*	
C19	0.2947 (7)	0.2284 (4)	0.7319 (7)	0.0363 (15)	
H20A	0.2877	0.0381	0.5581	0.055*	
H20B	0.4119	0.0503	0.6662	0.055*	
H20C	0.4187	−0.0043	0.5697	0.055*	
C20	0.3614 (9)	0.0155 (4)	0.6152 (7)	0.0436 (18)	
Hg1	0.02119 (3)	0.185765 (13)	0.82144 (2)	0.03426 (13)	
N1	0.1043 (6)	0.1013 (3)	0.9279 (6)	0.0395 (14)	
N2	0.2331 (6)	0.0113 (3)	0.9788 (6)	0.0385 (14)	
N3	−0.1082 (6)	0.2859 (3)	0.8103 (6)	0.0375 (13)	
N4	0.0466 (6)	0.2392 (3)	0.6444 (5)	0.0366 (13)	
O1	0.1436 (5)	0.2817 (3)	0.9589 (5)	0.0465 (13)	
O2	0.1103 (5)	0.3704 (3)	1.0576 (5)	0.0414 (12)	
H2	0.1924	0.3733	1.0740	0.050*	
O3	−0.1763 (5)	0.1700 (3)	0.9062 (5)	0.0412 (13)	
O4	−0.3977 (6)	0.1878 (3)	0.8430 (5)	0.0443 (13)	
O5	−0.1373 (5)	0.1400 (3)	0.6322 (5)	0.0380 (11)	
O6	−0.1182 (6)	0.1106 (3)	0.4498 (5)	0.0502 (15)	
O7	0.2823 (6)	0.1763 (3)	0.7841 (6)	0.0443 (13)	
O8	0.4098 (5)	0.2491 (3)	0.7194 (5)	0.0401 (12)	
H8	0.4604	0.2171	0.7225	0.048*	
O9	0.3170 (5)	−0.0289 (3)	0.6776 (5)	0.0415 (12)	
H9	0.2418	−0.0408	0.6411	0.050*	

### Atomic displacement parameters (Å^2^)

**Table:**

	*U*^11^	*U*^22^	*U*^33^	*U*^12^	*U*^13^	*U*^23^
C1	0.040 (4)	0.027 (3)	0.029 (3)	−0.005 (3)	0.000 (3)	−0.007 (3)
C2	0.041 (4)	0.040 (4)	0.031 (4)	0.001 (3)	−0.004 (3)	−0.002 (3)
C3	0.041 (4)	0.042 (4)	0.046 (4)	0.004 (3)	0.015 (3)	0.010 (3)
C4	0.029 (3)	0.042 (4)	0.034 (4)	0.000 (3)	0.001 (3)	0.007 (3)
C5	0.042 (4)	0.032 (4)	0.033 (4)	0.004 (3)	0.000 (3)	0.001 (3)
C6	0.027 (3)	0.039 (4)	0.044 (4)	0.005 (3)	0.001 (3)	0.008 (3)
C7	0.038 (4)	0.040 (4)	0.046 (4)	0.010 (3)	0.007 (3)	0.006 (3)
C8	0.034 (4)	0.044 (4)	0.042 (4)	−0.005 (3)	0.014 (3)	−0.002 (3)
C9	0.032 (4)	0.046 (4)	0.050 (5)	−0.005 (3)	0.016 (3)	−0.013 (4)
C10	0.038 (4)	0.051 (5)	0.035 (4)	0.007 (3)	0.013 (3)	0.007 (3)
C11	0.039 (4)	0.030 (3)	0.038 (4)	−0.005 (3)	0.014 (3)	0.009 (3)
C12	0.040 (4)	0.036 (4)	0.037 (4)	0.011 (3)	0.013 (3)	0.009 (3)
C13	0.034 (4)	0.038 (4)	0.026 (3)	0.011 (3)	0.011 (3)	0.003 (3)
C14	0.037 (4)	0.030 (3)	0.031 (4)	−0.004 (3)	−0.005 (3)	0.000 (3)
C15	0.045 (4)	0.043 (4)	0.030 (4)	0.003 (3)	0.015 (3)	0.004 (3)
C16	0.036 (4)	0.035 (4)	0.038 (4)	−0.008 (3)	0.013 (3)	−0.002 (3)
C17	0.031 (3)	0.020 (3)	0.040 (4)	0.003 (3)	0.004 (3)	0.002 (3)
C18	0.041 (4)	0.036 (4)	0.047 (4)	−0.012 (3)	0.011 (3)	0.002 (3)
C19	0.031 (3)	0.038 (4)	0.045 (4)	−0.005 (3)	0.018 (3)	0.005 (3)
C20	0.062 (5)	0.029 (3)	0.037 (4)	−0.011 (3)	0.008 (4)	−0.001 (3)
Hg1	0.02994 (18)	0.03898 (19)	0.03433 (18)	0.00717 (11)	0.00868 (11)	0.00914 (11)
N1	0.033 (3)	0.047 (4)	0.035 (3)	0.010 (3)	0.001 (3)	0.010 (3)
N2	0.035 (3)	0.045 (3)	0.034 (3)	−0.004 (3)	0.005 (3)	0.004 (3)
N3	0.044 (3)	0.037 (3)	0.034 (3)	0.006 (3)	0.014 (3)	0.003 (3)
N4	0.032 (3)	0.045 (3)	0.032 (3)	−0.007 (3)	0.007 (2)	0.000 (3)
O1	0.036 (3)	0.061 (4)	0.039 (3)	0.005 (3)	0.001 (2)	−0.007 (3)
O2	0.028 (3)	0.053 (3)	0.049 (3)	−0.004 (2)	0.020 (2)	−0.002 (3)
O3	0.042 (3)	0.044 (3)	0.041 (3)	0.012 (2)	0.017 (2)	0.024 (2)
O4	0.033 (3)	0.057 (4)	0.042 (3)	0.002 (2)	0.008 (2)	0.015 (2)
O5	0.031 (2)	0.044 (3)	0.038 (3)	0.002 (2)	0.006 (2)	0.008 (2)
O6	0.050 (3)	0.056 (3)	0.050 (3)	−0.030 (3)	0.023 (3)	−0.018 (3)
O7	0.039 (3)	0.036 (3)	0.056 (4)	−0.012 (2)	0.009 (3)	0.011 (2)
O8	0.029 (2)	0.053 (3)	0.040 (3)	−0.005 (2)	0.012 (2)	0.014 (2)
O9	0.044 (3)	0.050 (3)	0.035 (3)	−0.015 (2)	0.018 (2)	−0.006 (2)

### Geometric parameters (Å, °)

**Table:**

C1—C6	1.372 (10)	C13—O3	1.282 (9)
C1—C2	1.412 (11)	C14—N3	1.481 (9)
C1—N1	1.417 (9)	C14—C15	1.503 (11)
C2—C3	1.388 (10)	C14—H14A	0.9700
C2—H2A	0.9300	C14—H14B	0.9700
C3—C4	1.440 (10)	C15—N4	1.486 (10)
C3—H3	0.9300	C15—H15A	0.9700
C4—C5	1.361 (10)	C15—H15B	0.9700
C4—H4	0.9300	C16—C17	1.446 (9)
C5—C6	1.383 (10)	C16—N4	1.489 (9)
C5—H5	0.9300	C16—H16A	0.9700
C6—N2	1.389 (10)	C16—H16B	0.9700
C7—N1	1.348 (10)	C17—O5	1.248 (9)
C7—N2	1.364 (10)	C17—O6	1.271 (9)
C7—H7	0.9300	C18—C19	1.456 (11)
C8—N2	1.498 (10)	C18—N4	1.552 (9)
C8—C9	1.527 (10)	C18—H18A	0.9700
C8—H8A	0.9700	C18—H18B	0.9700
C8—H8B	0.9700	C19—O7	1.224 (9)
C9—C9^i^	1.509 (15)	C19—O8	1.293 (8)
C9—H9A	0.9700	C20—O9	1.291 (9)
C9—H9B	0.9700	C20—H20A	0.9822
C10—O1	1.148 (10)	C20—H20B	0.9735
C10—O2	1.316 (10)	C20—H20C	0.9611
C10—C11	1.536 (11)	Hg1—N1	2.138 (6)
C11—N3	1.492 (10)	Hg1—N4	2.364 (6)
C11—H11A	0.9700	Hg1—N3	2.390 (6)
C11—H11B	0.9700	Hg1—O3	2.473 (5)
C12—N3	1.488 (10)	Hg1—O5	2.547 (5)
C12—C13	1.569 (10)	Hg1—O1	2.604 (6)
C12—H12A	0.9700	O2—H2	0.8200
C12—H12B	0.9700	O8—H8	0.8200
C13—O4	1.258 (9)	O9—H9	0.8200
			
C6—C1—C2	124.2 (7)	C17—C16—N4	112.3 (6)
C6—C1—N1	109.3 (6)	C17—C16—H16A	109.1
C2—C1—N1	126.3 (6)	N4—C16—H16A	109.1
C3—C2—C1	113.6 (7)	C17—C16—H16B	109.1
C3—C2—H2A	123.2	N4—C16—H16B	109.1
C1—C2—H2A	123.2	H16A—C16—H16B	107.9
C2—C3—C4	121.8 (7)	O5—C17—O6	124.2 (6)
C2—C3—H3	119.1	O5—C17—C16	122.2 (6)
C4—C3—H3	119.1	O6—C17—C16	113.5 (6)
C5—C4—C3	121.9 (7)	C19—C18—N4	113.2 (6)
C5—C4—H4	119.1	C19—C18—H18A	108.9
C3—C4—H4	119.1	N4—C18—H18A	108.9
C4—C5—C6	116.7 (7)	C19—C18—H18B	108.9
C4—C5—H5	121.6	N4—C18—H18B	108.9
C6—C5—H5	121.6	H18A—C18—H18B	107.7
C1—C6—C5	121.6 (7)	O7—C19—O8	122.2 (7)
C1—C6—N2	106.0 (6)	O7—C19—C18	126.9 (7)
C5—C6—N2	132.4 (7)	O8—C19—C18	110.8 (6)
N1—C7—N2	110.3 (7)	O9—C20—H20A	111.4
N1—C7—H7	124.9	O9—C20—H20B	111.5
N2—C7—H7	124.9	H20A—C20—H20B	106.6
N2—C8—C9	111.6 (6)	O9—C20—H20C	111.3
N2—C8—H8A	109.3	H20A—C20—H20C	107.5
C9—C8—H8A	109.3	H20B—C20—H20C	108.3
N2—C8—H8B	109.3	N1—Hg1—N4	137.4 (2)
C9—C8—H8B	109.3	N1—Hg1—N3	146.4 (2)
H8A—C8—H8B	108.0	N4—Hg1—N3	75.6 (2)
C9^i^—C9—C8	113.0 (9)	N1—Hg1—O3	85.8 (2)
C9^i^—C9—H9A	109.0	N4—Hg1—O3	131.86 (19)
C8—C9—H9A	109.0	N3—Hg1—O3	68.26 (19)
C9^i^—C9—H9B	109.0	N1—Hg1—O5	106.7 (2)
C8—C9—H9B	109.0	N4—Hg1—O5	67.09 (18)
H9A—C9—H9B	107.8	N3—Hg1—O5	91.30 (19)
O1—C10—O2	123.9 (8)	O3—Hg1—O5	82.73 (18)
O1—C10—C11	124.8 (8)	N1—Hg1—O1	99.7 (2)
O2—C10—C11	111.3 (6)	N4—Hg1—O1	92.9 (2)
N3—C11—C10	108.2 (6)	N3—Hg1—O1	66.2 (2)
N3—C11—H11A	110.1	O3—Hg1—O1	100.25 (19)
C10—C11—H11A	110.1	O5—Hg1—O1	153.57 (18)
N3—C11—H11B	110.1	C7—N1—C1	105.6 (6)
C10—C11—H11B	110.1	C7—N1—Hg1	122.1 (5)
H11A—C11—H11B	108.4	C1—N1—Hg1	132.2 (5)
N3—C12—C13	110.5 (6)	C7—N2—C6	108.5 (6)
N3—C12—H12A	109.5	C7—N2—C8	126.6 (6)
C13—C12—H12A	109.5	C6—N2—C8	124.9 (6)
N3—C12—H12B	109.5	C14—N3—C12	108.5 (6)
C13—C12—H12B	109.5	C14—N3—C11	109.0 (5)
H12A—C12—H12B	108.1	C12—N3—C11	110.2 (6)
O4—C13—O3	124.0 (7)	C14—N3—Hg1	109.5 (4)
O4—C13—C12	117.8 (6)	C12—N3—Hg1	106.9 (4)
O3—C13—C12	118.1 (6)	C11—N3—Hg1	112.6 (4)
N3—C14—C15	111.8 (6)	C15—N4—C16	112.1 (6)
N3—C14—H14A	109.2	C15—N4—C18	109.8 (6)
C15—C14—H14A	109.2	C16—N4—C18	110.4 (6)
N3—C14—H14B	109.2	C15—N4—Hg1	108.6 (4)
C15—C14—H14B	109.2	C16—N4—Hg1	106.5 (4)
H14A—C14—H14B	107.9	C18—N4—Hg1	109.3 (4)
N4—C15—C14	112.7 (6)	C10—O1—Hg1	113.3 (6)
N4—C15—H15A	109.1	C10—O2—H2	109.5
C14—C15—H15A	109.1	C13—O3—Hg1	112.0 (4)
N4—C15—H15B	109.1	C17—O5—Hg1	108.7 (4)
C14—C15—H15B	109.1	C19—O8—H8	109.5
H15A—C15—H15B	107.8	C20—O9—H9	109.5
			
C6—C1—C2—C3	−1.8 (11)	O5—Hg1—N3—C14	−76.7 (5)
N1—C1—C2—C3	173.8 (7)	O1—Hg1—N3—C14	89.0 (5)
C1—C2—C3—C4	4.6 (11)	N1—Hg1—N3—C12	−83.0 (6)
C2—C3—C4—C5	−4.0 (12)	N4—Hg1—N3—C12	106.5 (4)
C3—C4—C5—C6	0.1 (11)	O3—Hg1—N3—C12	−41.0 (4)
C2—C1—C6—C5	−1.9 (11)	O5—Hg1—N3—C12	40.6 (4)
N1—C1—C6—C5	−178.2 (7)	O1—Hg1—N3—C12	−153.7 (5)
C2—C1—C6—N2	178.3 (7)	N1—Hg1—N3—C11	38.1 (7)
N1—C1—C6—N2	2.0 (8)	N4—Hg1—N3—C11	−132.3 (5)
C4—C5—C6—C1	2.7 (11)	O3—Hg1—N3—C11	80.1 (5)
C4—C5—C6—N2	−177.5 (8)	O5—Hg1—N3—C11	161.8 (4)
N2—C8—C9—C9^i^	−67.0 (10)	O1—Hg1—N3—C11	−32.5 (4)
O1—C10—C11—N3	−21.5 (10)	C14—C15—N4—C16	159.3 (6)
O2—C10—C11—N3	156.8 (6)	C14—C15—N4—C18	−77.6 (8)
N3—C12—C13—O4	168.5 (6)	C14—C15—N4—Hg1	41.9 (7)
N3—C12—C13—O3	−15.8 (9)	C17—C16—N4—C15	−72.5 (8)
N3—C14—C15—N4	−54.6 (8)	C17—C16—N4—C18	164.8 (6)
N4—C16—C17—O5	−16.4 (10)	C17—C16—N4—Hg1	46.2 (7)
N4—C16—C17—O6	159.9 (6)	C19—C18—N4—C15	165.0 (6)
N4—C18—C19—O7	−14.0 (12)	C19—C18—N4—C16	−71.0 (8)
N4—C18—C19—O8	161.4 (6)	C19—C18—N4—Hg1	45.9 (7)
N2—C7—N1—C1	−4.4 (8)	N1—Hg1—N4—C15	171.9 (5)
N2—C7—N1—Hg1	172.8 (5)	N3—Hg1—N4—C15	−15.9 (5)
C6—C1—N1—C7	1.4 (8)	O3—Hg1—N4—C15	26.0 (6)
C2—C1—N1—C7	−174.8 (7)	O5—Hg1—N4—C15	81.8 (5)
C6—C1—N1—Hg1	−175.4 (5)	O1—Hg1—N4—C15	−80.5 (5)
C2—C1—N1—Hg1	8.4 (11)	N1—Hg1—N4—C16	51.0 (6)
N4—Hg1—N1—C7	−12.1 (8)	N3—Hg1—N4—C16	−136.8 (5)
N3—Hg1—N1—C7	−178.3 (5)	O3—Hg1—N4—C16	−94.9 (5)
O3—Hg1—N1—C7	143.1 (6)	O5—Hg1—N4—C16	−39.1 (4)
O5—Hg1—N1—C7	62.0 (6)	O1—Hg1—N4—C16	158.6 (4)
O1—Hg1—N1—C7	−117.2 (6)	N1—Hg1—N4—C18	−68.3 (6)
N4—Hg1—N1—C1	164.2 (5)	N3—Hg1—N4—C18	103.8 (5)
N3—Hg1—N1—C1	−2.0 (9)	O3—Hg1—N4—C18	145.8 (4)
O3—Hg1—N1—C1	−40.6 (6)	O5—Hg1—N4—C18	−158.5 (5)
O5—Hg1—N1—C1	−121.7 (6)	O1—Hg1—N4—C18	39.3 (5)
O1—Hg1—N1—C1	59.1 (7)	O2—C10—O1—Hg1	174.3 (6)
N1—C7—N2—C6	5.8 (9)	C11—C10—O1—Hg1	−7.6 (10)
N1—C7—N2—C8	−177.3 (6)	N1—Hg1—O1—C10	−125.7 (6)
C1—C6—N2—C7	−4.7 (8)	N4—Hg1—O1—C10	95.2 (6)
C5—C6—N2—C7	175.6 (8)	N3—Hg1—O1—C10	22.3 (6)
C1—C6—N2—C8	178.3 (6)	O3—Hg1—O1—C10	−38.2 (6)
C5—C6—N2—C8	−1.4 (13)	O5—Hg1—O1—C10	56.1 (8)
C9—C8—N2—C7	100.6 (9)	O4—C13—O3—Hg1	153.3 (6)
C9—C8—N2—C6	−82.9 (9)	C12—C13—O3—Hg1	−22.1 (8)
C15—C14—N3—C12	−79.7 (7)	N1—Hg1—O3—C13	−166.5 (5)
C15—C14—N3—C11	160.2 (6)	N4—Hg1—O3—C13	−9.0 (6)
C15—C14—N3—Hg1	36.6 (7)	N3—Hg1—O3—C13	35.3 (5)
C13—C12—N3—C14	163.5 (6)	O5—Hg1—O3—C13	−59.1 (5)
C13—C12—N3—C11	−77.1 (7)	O1—Hg1—O3—C13	94.4 (5)
C13—C12—N3—Hg1	45.5 (6)	O6—C17—O5—Hg1	163.8 (6)
C10—C11—N3—C14	−80.9 (7)	C16—C17—O5—Hg1	−20.2 (8)
C10—C11—N3—C12	160.1 (6)	N1—Hg1—O5—C17	−101.6 (5)
C10—C11—N3—Hg1	40.9 (6)	N4—Hg1—O5—C17	33.4 (4)
N1—Hg1—N3—C14	159.6 (5)	N3—Hg1—O5—C17	107.1 (5)
N4—Hg1—N3—C14	−10.8 (4)	O3—Hg1—O5—C17	175.0 (5)
O3—Hg1—N3—C14	−158.4 (5)	O1—Hg1—O5—C17	76.6 (6)

Symmetry codes: (i) −*x*+1, −*y*, −*z*+2.

### Hydrogen-bond geometry (Å, °)

**Table:**

*D*—H···*A*	*D*—H	H···*A*	*D*···*A*	*D*—H···*A*
O2—H2···O5^ii^	0.82	1.74	2.534 (7)	164
O8—H8···O4^iii^	0.82	1.84	2.462 (7)	131
O9—H9···O6^iv^	0.82	2.00	2.744 (7)	150

Symmetry codes: (ii) *x*+1/2, −*y*+1/2, *z*+1/2; (iii) *x*+1, *y*, *z*; (iv) −*x*, −*y*, −*z*+1.
